# The Effect of Pomegranate Tea on the Clinical Outcomes and Symptom Alleviation in COVID‐19 Patients: A Double‐Blind Randomized Clinical Trial

**DOI:** 10.1155/cjid/3192659

**Published:** 2025-12-07

**Authors:** Vahid Reisi-Vanani, Fereidoun Rahmani, Soleiman Kheiri, Akbar Soleimani, Elham Bijad, Zohreh Abolhansanzadeh, Fatemeh Jamshidi-kia, Zahra Lorigooini

**Affiliations:** ^1^ Student Research Committee, Shahrekord University of Medical Sciences, Shahrekord, Iran, skums.ac.ir; ^2^ Department of Infectious Disease, School of Medicine, Hajar Hospital, Shahrekord University of Medical Sciences, Shahrekord, Iran, skums.ac.ir; ^3^ Modeling in Health Research Center, Shahrekord University of Medical Sciences, Shahrekord University of Medical Sciences, Shahrekord, Iran; ^4^ Department of Epidemiology and Biostatistics, School of Health, Shahrekord University of Medical Sciences, Shahrekord University of Medical Sciences, Shahrekord, Iran; ^5^ Department of Internal Medicine, School of Medicine, Hajar Hospital, Shahrekord University of Medical Sciences, Shahrekord, Iran, skums.ac.ir; ^6^ Medical Plants Research Center, Basic Health Sciences Institute, Shahrekord University of Medical Sciences, Shahrekord, Iran, skums.ac.ir; ^7^ Department of Horticulture Science, Faculty of Agriculture, Shahrekord University, Shahrekord, Iran, sku.ac.ir

**Keywords:** emerging communicable diseases, pomegranate, randomized controlled trial, SARS-CoV-2

## Abstract

**Introduction:**

COVID‐19, caused by SARS‐CoV‐2, emerged as a global pandemic in late 2019, leading to significant health and societal impacts. Treatment challenges have emerged as a result of viral mutations and the strain on healthcare resources, further intensified by the consequences of long‐term COVID‐19. Recent studies suggest pomegranate (*Punica granatum*) may offer immunomodulatory and antiviral benefits attributed to its polyphenol content. This study aimed to evaluate the impact of pomegranate tea on the clinical characteristics of COVID‐19 patients and investigate its potential therapeutic role.

**Methods:**

This double‐blinded randomized clinical trial evaluated the clinical characteristics of hospitalized COVID‐19 patients. Pomegranate was sourced and processed into a standardized aqueous extract, with a placebo prepared to match its appearance. Eligible participants with confirmed COVID‐19 pneumonia were randomized into intervention and placebo groups based on their respiratory support needs. Both groups received treatment during their hospital stay and continued postdischarge until symptom resolution. Standard treatment protocols were followed for all participants.

**Results:**

This double‐blinded randomized clinical trial evaluated the clinical characteristics of 66 hospitalized COVID‐19 patients. The median age of participants was 56 years, and demographics were similar across groups. Notably, 58.7% had symptom onset within the last 10 days. There were statistically significant differences in baseline laboratory markers or primary outcomes, including hospitalization duration and time to improvement. Secondary outcomes similarly demonstrated no significant differences in mortality, respiratory support duration, severity of clinical symptoms, or adverse effects.

**Conclusion:**

This study finds no significant advantage of pomegranate tea in managing COVID‐19 but highlights the inherent challenges in researching emerging infectious diseases. Our research highlights the necessity of continuously refining methodologies and adapting to the dynamic nature of emerging pathogens. Such efforts are essential for advancing the search for effective treatments and ultimately enhancing patient care. These findings contribute critically to the ongoing exploration of therapeutic strategies against viral infections, fostering a deeper understanding of potential interventions.

**Trial Registration:** Iranian Registry of Clinical Trials: IRCT20200416047104N1

## 1. Introduction

COVID‐19, caused by the novel SARS‐CoV‐2, emerged in late 2019 in Wuhan, China, and rapidly escalated into a global pandemic. The virus primarily spreads through respiratory droplets and contact with contaminated surfaces, leading to extensive spread through communities [1]. Epidemiological studies have shown that COVID‐19 can lead to a range of symptoms, from mild respiratory illness to severe complications, and that certain inhabitants, including the elderly and those with underlying diseases, are at higher risk of severe complications. Public health responses have included extensive screening, treating high‐risk patients, quarantining patients, and developing vaccines, all aimed at controlling the virus’s spread and mitigating its impact on healthcare systems and society. The pandemic has underscored the importance of global surveillance and collaboration in addressing infectious disease outbreaks [2].

The treatment of COVID‐19 has presented numerous challenges for healthcare systems worldwide, primarily due to the virus’s unpredictable nature and the variability in patient responses. Early in the pandemic, understanding the disease mechanisms and appropriate therapeutic interventions was limited, leading to a reliance on repurposed medications with mixed results [3]. As the virus mutates, variants like Delta and Omicron have exhibited differing levels of transmissibility and virulence, complicating treatment protocols [4]. The healthcare burden has also strained resources, creating disparities in access to treatments and intensive care across different regions. The emergence of long COVID has further complicated the landscape, necessitating ongoing research to address the lingering effects of the virus on patients. Moreover, the rapid pace of new data and evolving guidelines has made it challenging for healthcare professionals to stay updated, highlighting the need for robust support systems and education in managing this complex disease effectively [5]. This highlights the need to investigate new treatments and stay up‐to‐date alongside the virus development.

Pomegranate (*Punica granatum*) has garnered interest in recent studies for its potential immunomodulatory and antiviral properties, particularly in COVID‐19 [6, 7]. Rich in polyphenols, especially punicalagins and anthocyanins, pomegranate exhibits strong antioxidant properties that may help mitigate oxidative stress and inflammation, two factors commonly associated with severe COVID‐19 outcomes. Research has indicated that pomegranate extracts can inhibit viral replication and enhance immune response, potentially by modulating various signaling pathways [8]. Additionally, the fruit’s ability to influence the gut microbiome could further support overall health and immunity [9]. While these findings are promising, clinical studies are needed to establish the efficacy and mechanisms of pomegranate in preventing and treating COVID‐19. Herein, we have evaluated the effect of pomegranate on the clinical characteristics of COVID‐19 patients.

## 2. Methods

This double‐blinded randomized clinical trial was done in Hajar and Kashani hospitals in Shahrekord, Iran. The protocol of this study was approved by the Research Ethics Committees of Shahrekord University of Medical Sciences (IR.SKUMS.REC.1399.017).

### 2.1. Plant Preparation, Extraction, Formulation, and Blinding

The formulation was developed from the pomegranate fruit, including its septa and arils known for their rich polyphenolic and antioxidant contents. These parts are sourced from reputable centers. After confirmation of the samples by a botanist, the herbarium specimen was registered at The *Medical Plants Research Center* of SKUMS (No. 316). The aqueous extract was prepared using the maceration method, and the extract undergoes freeze‐drying. This pomegranate tea was formulated as a concentrated syrup, with each 10 cc containing 110 mg of pomegranate extract. A placebo was prepared using the same method but without pomegranate extract, incorporating red food coloring to standardize the appearance of the formulation. To ensure the blinding process, the placebo and drug were coded by pharmaceutical colleagues. The executing colleague, physician, and patient were unaware of the contents of each bottle. The executing colleague was instructed to provide both the drug and placebo in a ready‐to‐use form by dissolving 10 cc of the concentrated syrup in warm boiled water, which the patient should gargle before swallowing. Consequently, neither the patient nor the individual recording symptoms is informed about the contents of the tea.

### 2.2. Participants

Hospitalized male and nonpregnant females with the ability to understand and sign the written informed consent; positive SARS‐CoV‐2 real‐time PCR on upper respiratory swab; age ⩾ 18 years; SpO2 ⩽ 93%; pneumonia confirmed by chest imaging; and not taking medications like amitriptyline, codeine, desipramine, flecainide, fluoxetine, ondansetron, and tramadol (due to their interaction with pomegranate) were included in the study. We also did not include those individuals who were pregnant, lactating, or had a history of neurodegenerative conditions, dementia, or decompensated psychiatric disorder. The exclusion criteria were drug‐related hypersensitivity; chronic hepatic failure; chronic renal failure; use of any other investigational drug for COVID‐19 treatment; inability to drink and swallow drugs; transmission into another center; and ICU admission or any other condition that the patient–physician thinks could affect their safety or capability to get health services.

### 2.3. Intervention

Patients with the inclusion criteria were randomized into intervention and placebo groups based on their respiratory support. The randomization process was conducted using a stratified block randomization method, where patients were assigned to groups A and B in blocks of four, according to the respiratory support they received. Each block belonged to a specific respiratory support, with two participants randomly assigned to receive drug A and two to receive drug B. Both groups of patients received 10 cc of the concentrated syrup dissolved in warm boiled water four times daily. They will be instructed to gargle briefly before swallowing. The duration of consumption will coincide with the patient’s entire hospital stay, and after their discharge, the treatment will continue until symptoms improve. All patients in both groups received standard treatment for their condition.

It is important to note that while pomegranate is considered a food item, it has been included as a therapeutic supplement in this study. The dosage has been calibrated to match that of commercially available products.

### 2.4. Outcomes

The primary outcomes were improvement in 14 days, time to improvement, hospitalization duration, and time to deterioration. The secondary outcomes were death in 28 days, respiratory support duration, antiviral administration, corticosteroid administration, corticosteroid administration duration, antibiotic administration, ICU admission, and the need for home respiratory support.

### 2.5. Data Collection

Patients were visited daily during their hospitalization, and the researcher completed a checklist. The checklist consists of three sessions; the first one gathered comprehensive information about the patient, including demographic data, underlying health conditions, risk factors, symptoms at the time of presentation, and the method of COVID‐19 diagnosis. The second section assesses the presence and severity of symptoms using a four‐point scale (1—no symptoms, 2—mild, 3—moderate, and 4—severe) and records evidence of any medical complications, results of paraclinical tests, and medication information daily. The final section of the checklist pertains to details regarding the patient’s discharge process and any complications that arose during hospitalization. Additional variables to be measured include the duration of hospitalization and the length of time the patient received treatment until either death or transfer to an ICU. Each patient was followed after discharge by a daily telephone interview until the 14th day, and then again on the 28th day.

### 2.6. Safety and Adverse Events

No significant adverse effects have been reported regarding products derived from pomegranate, and potential drug interactions have been considered in the inclusion criteria for the study. However, their medication will be discontinued if patients experience any allergic reactions. Allergic responses such as itching, nasal discharge, or respiratory changes—similar to those observed with other medications—will also be monitored and documented. The safety of patients was assessed and recorded according to the criteria set forth by the NIH and the CTCAE Version 4 [10]. In addition to continuous clinical assessments, routine blood, urine, and stool tests were conducted, along with liver and kidney function evaluations. ECGs were also performed on the participants.

### 2.7. Statistical Analysis

Data were described using frequencies and percentages for qualitative variables, while means ± standard deviations were employed for quantitative variables with a normal distribution. For quantitative variables that did not follow a normal distribution, the median, along with the IQR, was reported. The normality of the distribution for each variable was assessed using the Kolmogorov–Smirnov test. Fisher’s exact or Chi‐square test was utilized to evaluate the differences between groups for qualitative variables. Independent *t*‐tests were applied to compare normally distributed quantitative variables, whereas the Mann–Whitney *U* test was used to compare non‐normally distributed quantitative variables. To analyze the severity of patients’ signs and symptoms during the study, the total severity of symptoms was calculated for each patient and compared between the two groups. A significance level of 0.05 was set for all tests, and statistical analyses were conducted using SPSS software (Version 22).

## 3. Results

**Figure 1:**
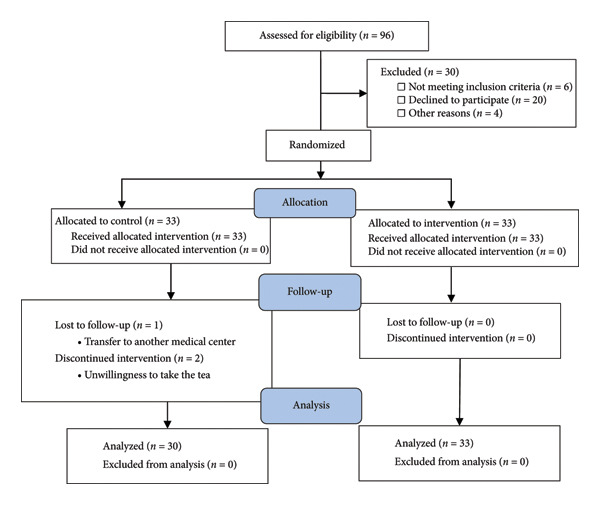
Consort flow diagram of the study population.

Sixty‐six patients were included in this study and were randomized to intervention and placebo groups in equal proportions. During the study, one patient was lost to follow‐up due to a volunteer transferring to another medical center, and two patients discontinued intervention due to their unwillingness to take the tea. Finally, 63 patients fulfilled the follow‐up period in the intervention (*N* = 33) and placebo (*N* = 30) groups (Figure 1). The median age of the patients was 56 (IQR, 44–66 years), and 52.4% were females. There were no statistical differences in demographic, clinical, and laboratory tests between the groups at baseline (Table 1). The time from symptom onset to randomization was below 10 days for 58.7% of patients, but for most patients in the placebo group (76.7%), symptoms began in less than 10 days.

**Table 1:** Clinical and demographic characteristics of patients at baseline.

Characteristic	Treatment	Placebo	*p* value
Age (years)	59 (46.5–66.0)	52 (42.0–67.0)	0.31
Sex (Male)	15 (45.5%)	15 (50.0%)	0.80
Time from symptom onset to randomization			
• Lower than 10 days	14 (42.4%)	23 (76.7%)	0.01
• More than 10 days	19 (57.6%)	7 (23.3%)	
Smoking (Yes)	4 (12.1%)	5 (16.7%)	0.72
Coexisting conditions			
• Diabetes	4 (12.1%)	8 (26.7%)	0.20
• Cardiovascular disease	14 (42.4%)	10 (33.3%)	0.60
• Pulmonary disease	14 (42.4%)	8 (26.7%)	0.29
• Malignancies	1 (3%)	2 (6.7%)	—
• Renal disease	2 (6.1%)	2 (6.7%)	1.0
• Hepatic disease	14 (42.4%)	6 (20.0%)	0.06
• Rheumatologic disease	3 (9.1%)	1 (3.1%)	0.61
• Hematologic disease	7 (21.2%)	9 (30.0%)	0.56
• Psychologic disease	2 (6.1%)	5 (16.7%)	0.24
Corticosteroid before enrolment	0 (0.0%)	3 (10.0%)	0.10
Antiviral before enrolment	4 (12.1%)	2 (6.7%)	0.67
Respiratory support at randomization			
• No support	8 (24.2%)	7 (23.3%)	0.993
• Low flow	23 (69.7%)	21 (70.0%)	
• High flow	2 (6.1%)	2 (6.7%)	

Evaluating laboratory markers of patients included in the study indicated no significant difference in lymphocytes, platelets, CRP, Ferritin, ESR, LDH, and CPK. Our evaluation revealed that a higher number of patients with leukopenia were assigned to the placebo group (*p* = 0.02) (Table 2).

**Table 2:** Laboratory characteristics of patients at baseline.

Lab data	Treatment	Placebo	*p* value
White‐cell count (× 10^9/*L*^)			
• Less than 4000	2 (6.1%)	10 (33.3%)	0.02
• 4000–10000	30 (90.9%)	19 (63.3%)	
• More than 10,000	1 (3.0%)	1 (3.3%)	
Lymphocyte count			
• < 1.0 × 10^9/*L*^	15 (45.5%)	9 (30.0%)	0.29
• ≥ 1.0 × 10^9/*L*^	18 (54.5%)	21 (70.0%)	
Platelet count (× 10^9/*L*^)			
• < 100 × 10^9/*L*^	1 (3.0%)	2 (6.7%)	0.60
• ≥ 100 × 10^9/*L*^	32 (97.0%)	28 (93.3%)	
CRP	10.0 (5.0–16.0)	7.0 (4.0–10.0)	0.09
Ferritin	287.0 (124.0–737.0)	350.0 (155.0–714.75)	0.65
ESR	36.0 (25.75–57.5)	28.0 (16.5–38.0)	0.012
LDH	418.0 (322.5–684.5)	379.0 (321.0–504.75)	0.11
CPK	91.0 (63.2–144.5)	78.0 (35.0–117.0)	0.34

*Note:* Lab, laboratory; CRP, C‐reactive protein; LDH, lactate dehydrogenase; CPK, creatine kinase.

Abbreviation: ESR, erythrocyte sedimentation rate.

### 3.1. Primary Outcomes

Patients assigned to the pomegranate tea group showed no statistically significant difference from those assigned to the placebo group in terms of improvement over 14 days, deterioration, hospitalization days, or time to improvement and deterioration (Table 3).

**Table 3:** Clinical primary outcomes of patients in the study groups.

Outcome	Treatment	Placebo	*p* value
Improvement in 14 days	28 (84.8%)	25 (83.3%)	1.0
Time to improvement (days)	6 (4–8)	5 (4–8)	0.83
Hospitalization (days)	6 (4–8)	5 (4–8)	0.87
Deterioration	6 (18.2%)	6 (20.0%)	1.0
Time to deterioration (days)	2 (1.75–4.25)	2 (1.0–2.5)	0.40

### 3.2. Secondary Outcomes

Evaluating secondary outcomes of the study also indicated no significant difference in mortality on day 28, respiratory support duration, antiviral administration, corticosteroid administration, corticosteroid administration duration, antibiotic administration, ICU admission, and the need for respiratory support after discharge (Table 4).

**Table 4:** Clinical secondary outcomes of patients in the study groups.

Outcome	Treatment	Placebo	*p* value
Death at 28 days	6 (18.2%)	2 (6.7%)	0.26
Respiratory support day	4.00 (2.75–7.00)	4.00 (3.00–7.00)	0.75
Antiviral administration	31 (93.9%)	28 (93.3%)	1.0
Corticosteroid administration	13 (39.4%)	9 (30.0%)	0.59
Corticosteroid administration day	5.50 (1.00–13.00)	4.00 (3.00–9.00)	0.63
Antibiotic administration	27 (81.8%)	20 (66.7%)	0.31
ICU admission	5 (15.2%)	4 (13.3%)	1.0
Home respiratory support			
• NO respiratory support	13 (39.4%)	13 (43.3%)	0.59
• Mild	16 (48.5%)	13 (43.3%)	
• Moderate	1 (3.0%)	0 (0.0%)	

As explained in the Methods section, we have scaled the severity of patients’ signs and symptoms. Each clinical symptom was assessed for 14 days, and for most of them, the severity of symptoms was documented in four‐point scale (1—no symptoms, 2—mild, 3—moderate, 4—severe). Statistical analysis of the sum of the data and the result of their difference is as follows:

Severity of cough (*p* = 0.42), frequency of cough (*p* = 0.76), severity of myalgia (*p* = 0.24), severity of fatigue (*p* = 0.51), severity of fever (*p* = 0.78), severity of thirst (*p* = 0.57), severity of dyspnea (*P* = 0.89), severity of sore throat (*p* = 0.16), severity of chills (*p* = 0.39), severity of confusion (*p* = 089), blood oxygen saturation (*p* = 0.75), severity of oxygen therapy (*p* = 0.75), fever duration (*p* = 0.80), constipation (*p* = 0.71), and diarrhea (*p* = 0.10). Evaluating adverse events and intervention safety indicated no significant difference between intervention and placebo. The detailed distribution of these parameters is presented in Supporting Table 1 (see Supporting Information).

### 3.3. Day 14 Laboratory Results

On the 14th day of treatment, laboratory markers were reassessed to determine any possible biochemical effects of the intervention (Table 5). Both groups exhibited modest normalization trends in inflammatory and enzymatic parameters compared to baseline; however, there were no significant differences in CRP, ferritin, ESR, LDH, or CPK levels between groups (*p* > 0.05). The distribution of white blood cell counts varied slightly between groups (*p* = 0.019), with a greater percentage of patients in the pomegranate group exhibiting counts > 10 × 10^9/*L*^ (72.7% vs. 0%). In contrast, the majority of patients receiving a placebo had counts within the normal range (4000–10,000 × 1010^9/*L*^). Although there was a variation in the distribution of white‐cell counts (*p* = 0.019), no significant differences were observed in clinical outcomes or symptom severity, indicating no clinically meaningful hematologic effect.

**Table 5:** Laboratory characteristics of patients on the 14th day of intervention.

Lab data	Treatment	Placebo	*p* value
White‐cell count (× 10^9/*L*^)			
• Less than 4000	—	2 (6.7%)	
• 4000–10000	4 (12.1%)	17 (56.7%)	0.019
• More than 10,000	24 (72.7%)	—	
Lymphocyte count			
• < 1.0 × 10^9/*L*^	20 (60.6%)	15 (50%)	0.61
• ≥ 1.0 × 10^9/*L*^	5 (15.2%)	4 (13.3%)	
Platelet count (× 10^9/*L*^)			
• < 100 × 10^9/*L*^	1 (3%)	—	0.57
• ≥ 100 × 10^9/*L*^	22 (66.7%)	17 (56.7%)	
CRP	6.50 (4.50–12.32)	5.00 (2.25–8.25)	0.67
Ferritin	217.0 (119.45–360.15)	283.0 (105.87–638.52)	0.19
ESR	42.00 (20.25–53.50)	30.00 (22.00)	0.93
LDH	359.00 (274.00–412.00)	318.00 (274.00–349.00)	058
CPK	50.00 (43.27–97.00)	54.00 (29.00–83.00)	0.31

*Note:* Lab, laboratory; CRP, C‐reactive protein; LDH, lactate dehydrogenase; CPK, creatine kinase.

Abbreviation: ESR, erythrocyte sedimentation rate.

### 3.4. Adverse Events and Safety

An analysis of adverse events revealed no significant safety issues associated with the use of pomegranate tea (Table 6). The rates of gastrointestinal, cardiovascular, hematologic, and respiratory complications were comparable across groups. None of the events achieved statistical significance (*p* > 0.05), despite some (such as liver dysfunction, anemia, ARDS, and superinfection) being numerically more common in the intervention group. Crucially, there were no reports of allergic or hypersensitive reactions, indicating that the formulation is safe.

**Table 6:** Adverse events during the study in intervention and placebo groups.

	Treatment	Placebo	*p* value
Superinfection	6 (18.2%)	8 (26.7%)	0.35
ARDS	5 (15.2%)	1 (3.3%)	0.26
Pneumothorax	1 (3.0%)	0 (0.0%)	1.0
Plural effusion	3 (9.1%)	0 (0.0%)	0.24
Bronchiolitis	16 (48.5%)	10 (33.3%)	0.58
Encephalitis	1 (3.0%)	0 (0.0%)	1.0
Stroke	2 (6.1%)	0 (0.0%)	0.49
HF	1 (3.0%)	1 (3.3%)	1.0
Myocarditis	1 (3.0%)	0 (0.0%)	1.0
Arrhythmia	2 (6.1%)	0 (0.0%)	0.49
Cardiac ischemia	1 (3.0%)	0 (0.0%)	1.0
Bacteremia	2 (6.1%)	0 (0.0%)	0.49
Coagulopathy	2 (6.1%)	0 (0.0%)	0.49
Anemia	9 (27.3%)	11 (36.7%)	0.41
ARI	3 (9.1%)	5 (16.7%)	0.44
GI	12 (36.4%)	6 (20.0%)	0.18
Pancreatitis	1 (3.0%)	0 (0.0%)	1.0
Liver dysfunction	5 (15.2%)	2 (6.7%)	0.44
Hyperglycemia	10 (30.3%)	9 (30.0%)	0.34
Hypoglycemia	2 (6.1%)	2 (6.7%)	1.0
Hypertension	4 (12.1%)	0 (0.0%)	0.08
Hypotension	1 (3.0%)	0 (0.0%)	1.0

*Note:* GI, gastrointestinal side effects.

Abbreviations: ARDS, acute respiratory distress syndrome; HF, heart failure; AKI, acute kidney injury.

## 4. Discussion

Our study, which investigated the effects of pomegranate tea added to standard care, found no association with clinical improvement or mortality in COVID‐19 patients admitted to the hospital. The impact on primary outcomes, including 28‐day mortality, improvement within 14 days, deterioration, hospitalization days, time to improvement, and deterioration in COVID‐19 patients, revealed no statistically significant differences between the intervention and placebo groups. Our investigation on secondary outcomes and adverse events also revealed no significant difference between groups. This outcome raises important questions about the therapeutic potential of many suggested agents and medicines in the context of emerging diseases. While pomegranate has been recognized for its antioxidant and anti‐inflammatory properties, which could theoretically enhance immune function and are suspected to have an antiviral effect through blocking the virus S‐glycoprotein‐ACE2 contact [7, 11, 12], our findings suggest that RCTs play a critical role in evidence‐based medicine, particularly as we have experienced in the COVID‐19 pandemic. Relying exclusively on in vitro, in vivo, and in silico models, such as the experience of excessive administration of hydroxychloroquine, may result in misinformed clinical practices; therefore, obtaining clinical evidence from evidence‐based medicine studies must play a significant role in clinical practice [12, 13].

A critical challenge in designing RCTs for infectious diseases, particularly emerging ones like COVID‐19, is the heterogeneity in disease presentation and progression among patients. The course of the disease can vary widely, with some individuals experiencing mild symptoms while others progress rapidly to severe illness [14]. This variability complicates the identification of appropriate endpoints and may obscure the efficacy of interventions that could be beneficial for specific subgroups [15]. Furthermore, the timing of intervention is crucial; administering a treatment during a critical phase of disease progression may yield different results than when given at an earlier or later stage [16]. This heterogeneity could also affect the main body of an RCT, specifically the process of randomization. In this study, we assigned patients to groups using a stratified block randomization method based on their respiratory support [17]. Unfortunately, some other variants indicating the course of the disease, such as the time from symptom onset to randomization, were not similar in both groups, which could affect the study’s results. In our study, more patients with leukocytosis were assigned to the placebo group, which aligns with the observed difference in disease progression between our groups, given that COVID‐19 patients with leukopenia or leukocytosis tend to experience more severe disease [18]. Still, statistical analysis revealed no significant effect of the cofounding factor “Time from symptom onset to randomization” on the study’s primary and secondary outcomes.

Additionally, the rapid evolution of emerging pathogens poses further complications in clinical trial design. The emergence of new variants can alter disease severity, transmissibility, and treatment response, thereby impacting the generalizability of study findings [19]. One possible solution for this problem might be adaptive trial designs that allow flexibility in response to emerging data during the pandemic, optimizing resource use and accelerating the evaluation of therapeutic interventions. This could enhance trial efficiency and improve the likelihood of identifying effective treatments for COVID‐19 or other diseases [20].

Recent in silico and in vitro studies have demonstrated that key polyphenols in *Punica granatum*, particularly punicalagin, punicalin, and ellagic acid, can inhibit the binding of the SARS‐CoV‐2 spike protein to the ACE2 receptor and exert notable anti‐inflammatory effects [6, 7, 21]. However, the phytochemical composition of herbal preparations varies depending on plant part, extraction procedure, and cultivar, emphasizing the need for standardized extracts quantified for key marker compounds to ensure reproducibility and comparability across clinical trials [22]. Accordingly, our randomized controlled trial provides complementary clinical data to the existing mechanistic evidence, illustrating that biological activity observed in laboratory models does not necessarily translate into clinical efficacy. Future investigations should employ chemically standardized pomegranate extracts to elucidate their therapeutic potential against viral infections [23].

In conclusion, while our RCT did not demonstrate a significant benefit of pomegranate tea in managing COVID‐19, it underscores the complexities inherent in studying infectious diseases. As we continue to explore potential therapeutic options, refine our methodologies, and adapt to the evolving landscape of emerging pathogens, it is crucial. This will ultimately enhance our ability to identify effective treatments and improve patient outcomes.

**Nomenclature**
CTCAE: Common Terminology Criteria for Adverse Events
COVID‐19: Coronavirus disease
CRP: C‐reactive protein
CPK: Creatine kinase
ECGs: Electrocardiograms
ESR: Erythrocyte sedimentation rate
ICU: Intensive care unit
IQR: interquartile range
LDH: Lactate dehydrogenase
NIH: National Institutes of Health
SpO2: Peripheral oxygen saturation
RCT: Randomized controlled trial
S‐glycoprotein‐ACE2: S‐glycoprotein‐angiotensin‐converting enzyme 2

## Ethics Statement

This research was carried out in accordance with the principles established in the Declaration of Helsinki and followed the guidelines of Good Clinical Practice (GCP). Approval from the Ethics Committee of Shahrekord University of Medical Sciences was secured before the trial commenced (IR.SKUMS. REC.1399.017). All participants gave their written informed consent before joining the study.

## Conflicts of Interest

The authors declare no conflicts of interest.

## Author Contributions

Vahid Reisi‐Vanani, Freidoun Rahmani, Akbar Soleimani, Elham Bijad, Zohreh Abolhansanzadeh, and Fatemeh Jamshidi‐kia performed the study and wrote the paper. Soleiman Kheiri conceived and designed the study; analyzed and interpreted the data; and wrote the paper. Zahra Lorigooini conceived and designed the study; contributed reagents, materials, and analysis data; performed the study; and wrote the paper.

## Funding

This study was supported by a research grant (5361) from Shahrekord University of Medical Sciences, Shahrekord, Iran.

## Supporting Information

The supporting file contains Supporting Table 1, which provides detailed daily symptom severity scores and additional clinical data supporting the main findings of the study.

## Supporting information


**Supporting Information** Additional supporting information can be found online in the Supporting Information section.

## Data Availability

Data are available upon request to authors.
