# Supplementary material for: The Effect of Pomegranate Tea on the Clinical Outcomes and Symptom Alleviation in COVID‐19 Patients: A Double‐Blind Randomized Clinical Trial
Source: Can J Infect Dis Med Microbiol. 2025 Dec 7;2025:3192659. doi: 10.1155/cjid/3192659 (PMC12747076; doi:10.1155/cjid/3192659)
Supplement: Supplementary file 1 — Supporting Information Additional supporting information can be found online in the Supporting Information section. [file CJID-2025-3192659-s001.docx]

**Supplementary Material**

Supplementary Table 1. The sum of the severity of patients' signs and symptoms during 14 days in the study groups.

| Signs or symptoms | Treatment | Placebo | P-Value |
| --- | --- | --- | --- |
| Severity of Cough | 19 (15 – 28.5) | 19.5 (14-23.25) | 0.42 |
| Frequency of cough | 0 (0-2.5) | 0 (0 – 3.5) | 0.76 |
| Severity of myalgia | 16 (13-23.5) | 14 (14-17.25) | 0.24 |
| Severity of fatigue | 21 (14.5-28.5) | 17.5 (14-25.25) | 0.51 |
| Severity of fever | 14 (14-16) | 14 (10.75-16) | 0.78 |
| Severity of thirst | 18 (14-27) | 17 (14-21.75) | 0.57 |
| Severity of dyspnea | 15 (14-18.5) | 14 (11.75-21.75) | 0.89 |
| Severity of sore throat | 14 (12.5-15.5) | 14 (10.75-14) | 0.16 |
| Severity of chills | 14 (13-16.5) | 14 (11-15.25) | 0.39 |
| Severity of confusion | 14 (14-17.5) | 14 (10.75-15) | 0.89 |
| Blood oxygen saturation | 467 (282-653) | 429 (276.75-789.5) | 0.75 |
| Severity of oxygen therapy | 8 (4.5-13) | 9.5 (5.75-13.5) | 0.75 |
| Fever duration | 0 (0-1) | 0 (0-0) | 0.80 |
| Constipation | 1 (1-1.5) | 1 (1-1.25) | 0.71 |
| Diarrhea | 1 (1-2) | 1 (1-1) | 0.10 |
